# A case of syphilis associated with immune reconstitution inflammatory syndrome and review of the literature

**DOI:** 10.1186/s12981-023-00522-2

**Published:** 2023-05-11

**Authors:** Luca Pipitò, Alice Annalisa Medaglia, Marcello Trizzino, Silvia Bonura, Claudia Gioè, Paola Di Carlo, Claudia Colomba, Antonio Cascio

**Affiliations:** 1 Infectious and Tropical Diseases Unit- Department of Health Promotion, Mother and Child Care, Internal Medicine and Medical Specialties “G D’Alessandro”, University of Palermo, Palermo, Italy; 2 Infectious and Tropical Disease Unit, AOU Policlinico “P. Giaccone”, Via del Vespro 129, 90127 Palermo, Italy; 3 Pediatric Infectious Diseases Unit, ARNAS Civico-Di Cristina-Benfratelli Hospital, 90127 Palermo, Italy; 4 Palermo Fast-Track City, Casa Dei Diritti, Via Libertà 45, 90143 Palermo, Italy

**Keywords:** HIV, IRIS, Syphilis, Paradoxical, Unmasking

## Abstract

**Background:**

Immune reconstitution inflammatory syndrome (IRIS) associated with syphilis has rarely been described in HIV-infected patients. Diagnosis can be challenging because it is not always possible to discern it from a recent infection or a worsening of an undiagnosed one.

**Case presentation:**

An HIV-positive 42-year-old man with a poor compliance history of antiretroviral therapy presented at our unit and complained of ocular symptoms. Ocular syphilis diagnosis was posed after initial misdiagnosing with cytomegalovirus infection, and antiretroviral therapy compliance improved after switching to a bictegravir-based regimen. Despite intravenous (IV) penicillin, we observed an initial worsening with the appearance of new skin lesions, and IRIS syphilis was suspected. In the literature, 14 cases of IRIS syphilis are described, all regarding male patients. Seven were HIV naïve to therapy, and 7 HIV-experienced with poor therapy compliance. Basal syphilis serology was negative in ten, with subsequent seroconversion after the development of IRIS. IRIS-syphilis development was observed after a median time of 28 days from ART initiation; 10 cases were considered "unmasking-IRIS" and 4 "paradoxical-IRIS". Skin and ocular involvement were the most often reported. In most cases, it was not necessary to use a systemic steroid. A good outcome was reported in 12.

**Conclusions:**

Syphilis should be considered in differential diagnosis with other diseases associated with IRIS. A negative syphilis serology before beginning antiretroviral therapy could convey the impression that syphilis has been ruled out. Whereas a high index of suspicion should be maintained when symptoms suggestive of syphilis, such as ocular and skin manifestations, are noticed after therapy has begun.

## Background

Immune reconstitution inflammatory syndrome (IRIS) is a condition during the clinical course of HIV infection in which there is a paradoxical worsening or new onset of opportunistic infections in an HIV-positive patient following the initiation of antiretroviral therapy (ART) or switching to more potent ART regimen [1]. “Unmasking IRIS” is defined as a new appearance of symptoms related to an unknown infection, and “paradoxical IRIS” is defined as the worsening of a previously noted [2]. IRIS has rarely been reported in the context of syphilis infection [3].

## Case presentation

An HIV-positive 42-year-old man with a history of poor compliance to antiretroviral therapy presented in February 2022 with blurry vision, ocular pain, and photophobia in the left eye. He denied sexual encounters except oral in the past six months. He was on ART with boosted darunavir, tenofovir alafenamide and emtricitabine, but he was not taking the therapy regularly. CD4 count was 196 cells/μl (9%), and HIV viral load was 258 copies/ml. Left eye examination showed uveitis, and ocular cytomegalovirus infection was suspected. Valganciclovir was promptly started, and ART was switched to a bictegravir-based regimen with adherence improvement. Two weeks later, he presented with worsening symptoms and complaints of contralateral eye involvement and was hospitalized. His blood exams were unremarkable, except for C reactive protein (15 mg/l – normal <5) on admission. Specific luetic serology was positive with rapid plasma reagin (RPR) 1:32 and a precedent negative, while cytomegalovirus viral load on blood was negative. Valganciclovir was discontinued, and Penicillin G 3 million units iv q4h plus ocular steroid were administered because of ocular involvement. A cerebral CT scan and MRI highlighted bilateral sclera enhancement. Lumbar puncture showed 130 cells/μl, increased cerebrospinal fluid (CSF) total protein levels (729 mg/dl, range 150-450 mg/dl), and the glucose value was normal.

Polymerase chain reaction for Treponema pallidum and RPR on liquor were both negative. Despite the therapy for syphilis, during the first week, he experienced a worsening of general clinical conditions associated with an increase in CD4 count (318 cells/μl) and a reduction in HIV-RNA (42 cp/ml). He developed desquamating papules on the scalp, back, soles and palms, patchy alopecia, eyebrow loss and the appearance of neck, axillary and inguinal lymphadenopathy. In the following days, the patient improved; he had ocular symptoms resolution at six months from discharge.

## Discussion and conclusions

We considered our case a “paradoxical IRIS” because we observed a worsening of symptoms and the appearance of new lesions. At the first presentation, the worsening of symptoms with the involvement of the contralateral eye was related to the misdiagnosis. The second time the patient was on specific therapy with IV penicillin instead. The condition resolved without the necessity of steroids or other anti-inflammatory drugs.

The definition of IRIS-syphilis is complex as it is not always possible to discern it from a recent infection or a worsening of an undiagnosed one. Syphilis syndromes have rarely been described in the context of immune reconstitution, and only a few cases have been reported in the literature [3]. A computerized search was performed without language restriction using PubMed, SCOPUS and Web of Science™ for all cases of IRIS-syphilis from database inception until April 2022.

**Fig.1:**
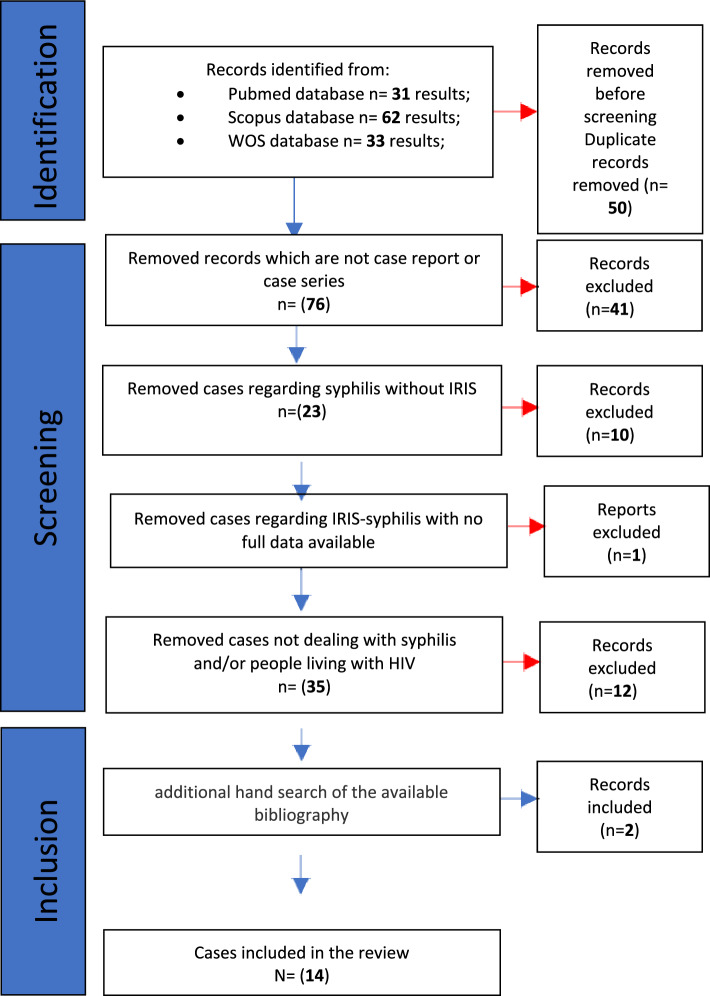
Research of literature: identification of cases, screening, and inclusion

Our review using PubMed, SCOPUS and Web of Science™ (Figure 1) showed only 14 cases of IRIS associated with syphilis described in the literature. The cases were divided into "unmasking syphilis", characterized by a new appearance of syphilis symptoms after ART initiation, and "paradoxical syphilis", characterized by worsening previously present symptoms.

Epidemiological data and HIV status information are analytically shown in Table 1. Clinical characteristics of all the patients retrieved are analytically illustrated in Table 2, and investigations during IRIS, therapy and outcome are shown in Table 3. All patients were male with a 43-year-old median age (IQR 36 to 46). Of these cases, seven were concerned with HIV naïve to therapy patients and 7 with HIV infection known for years with poor compliance to ART. The HIV viral load at baseline was reported in 12 cases with a median value of 177,828 copies/ml (IQR 71,000 to 280,000 copies/ml), and CD4+ T lymphocytes count was reported in 14 cases prevalently lower than 200, with a median value of 91 cells/μl (interquartile range 25.5 to 163.5 cells/μl). The IRIS manifestations were associated with a reduction of HIV viral load by approximately three logarithms and to increase in CD4+ cell count.

**Table 1:** Epidemiological data and pre-IRIS data

Author, country, and years of publication	Sex	Age (years)	CDC	Naïve or experienced to ART	HIV viral load (copies/ml) and CD4 + count (cells/μl)	Basal treponemal/non treponemal test	Precedent sexual encounters (last six months)
Mitra et al. [3], India, 2021	M	48	C3	Naïve	300,000/20	NR^*1^/Negative	No
Alcedo et al. [8], Peru, 2019	M	21	A3	Naïve	96,000/91	NR/Negative	Not reported
Yap et al. [7], Australia, 2017	M	71	B3	Naïve	177,828 /185	Negative/Negative	Yes, four weeks ago
Frunza-Stefan et al. [11], China, 2016	M	52	B3	Naïve	280,000 /40	NR/Negative	Yes, six months ago
Braue et al.[4], USA, 2015	M	36	NR	Experienced	NR/324	NR/NR	NR
Khatri et al. [12], USA, 2014	M	43	C3	Experienced	344,000/42	NR/RPR 1:16	No
Vasudevan et al. [9], India, 2013	M	44	C3	Naïve	200,000/92	NR/Negative	Yes, six months ago
Bernal et al. [13], Spain, 2009	M	43	B2	Experienced	31,500/320	NR/Negative	NR
Hardwick et al. [14], England, 2006	M	36	NR	Experienced	NR/NR	Negative /Negative	NR
Moloney et al. [15], Australia, 2004	M	45	A3	Experienced	NR/12	Negative/Negative	Yes
Brochard et al. [16], France, 2017	M	24	C3	Experienced	251,189/31	Negative/Negative	NR
Ceccarelli et al. [17], Italy, 2010	M	28	A3	Naïve	100,000/142	Negative/Negative	NR
Rushing et al. [5], USA, 2008	M	46	C3	Naïve	71,000/8	NR/RPR 1:256	NR
Bucher et al.[6], USA, 2011	M	46	NR	Experienced	25,300/106	Positive/RPR 1:1024	NR
Our case, Italy, 2022	M	42	C3	Experienced	258/196	Negative/Negative	Yes

^*^^1^not reported

**Table 2:** Type of IRIS and clinical manifestation

Author	Type of Iris and Neurosyphilis/ ocular syphilis (yes or no)	Time to IRIS after starting ARVT (days)	Cutaneous manifestation (yes or no)	Genital lesion (yes or no)	Ocular manifestation (yes or no, type)	Lymphadenopathy (yes or no)	Systemic symptoms (yes or no)	Neurological symptoms (yes or no, type)	Other manifestation (yes or no, type)
Mitra et al. [3]	Unmasking, no	42	Yes	Yes	No	No	No	No	No
Alcedo et al. [8]	Paradoxical, yes	15	Yes	Yes	Yes, panuveitis	No	No	Yes, headache	No
Yap et al. [7]	Unmasking, yes	14	Yes (lues maligna)	No	Yes, vitritis	No	Yes	No	No
Frunza-Stefan et al. [11]	Unmasking, no	3	Yes	No	No	No	No	No	No
Braue et al.[4]	Unmasking, no	14	Yes, (lues maligna)	No	No	Yes	Yes	No	Yes, rectal mass
Khatri et al. [12]	Unmasking, no	28	Yes	No	No	No	No	No	No
Vasudevan et al. [9]	Unmasking, no	60	Yes	No	No	No	No	No	No
Bernal et al. [13]	Unmasking, yes	30	No	No	Yes, panuveitis	No	Yes	No	No
Hardwick et al. [14]	Paradoxical, yes	60	Yes	No	Yes, iritis	No	Yes	No	Yes, rhinitis
Moloney et al. [15]	Unmasking, yes	540	No	No	Yes, scleritis, retinitis and vitritis	Yes	No	Yes, headache	No
Brochard et al. [16]	Unmasking, no	3	Yes	No	No	No	Yes	No	Yes, left knee arthritis
Ceccarelli et al. [17]	Unmasking, yes	28	No	No	Yes, uveo-papillitis, retinal detachment	No	No	Yes, headache	No
Rushing et al. [5]	Paradoxical, yes	42	Yes	No	No	No	No	Yes, headache, word-finding difficulty and arm numbness	No
Bucher et al.[6]	Paradoxical, yes	10	Yes	No	No	No	No	Yes, meningovascular syphilis (headache, photophobia, ataxia, memory impairment and diplopia)	No
Our case	Paradoxical, yes	21	Yes	No	Yes, retinitis, iritis and vitritis	Yes	No	No	No

**Table 3:** Investigations during IRIS, therapy, and outcome

Author	HIV viral load during IRIS (copies/ml)	CD + Count during IRIS (cells/mm^3^)	Syphilis serology during IRIS	Spinal tap and CSF analysis (yes or no, normal or alteration type, syphilis test)	Skin biopsy (yes or no), immunostaining (positive or negative, and syphilis PCR on	Other invasive exams	Imaging	Syphilis therapy	IRIS therapy (yes or no and type)	Outcome
			(treponemal/non treponemal)		samples		(yes or no and type)			
Mitra et al. [3]	0	160	Positive/Negative	Yes, normal, serology negative, PCR not performed	Yes, immunostaining and PCR not performed	No	No	three weekly doses of injection benzathine penicillin 2.4 MU	No	Good clinical outcome
Alcedo et al. [8]	453	140	NR^*1^/Positive	Yes, normal, VDRL 1:2, PCR not performed	Yes, immunostaining and PCR not performed	No	No	IV penicillin G sodium 16 MU/day for 14 days	Yes, corticosteroid therapy for 14 days	Good clinical outcome
Yap et al. [7]	65	252	Positive/Positive	No	Yes, immunostaining not performed and PCR positive	Vitreous biopsy, PCR positive	No	IV benzylpenicillin 1.8 g q.i.d. for 15 days in addition to 60-mg prednisolone for 5 days to prevent a Jarisch–Herxheimer reaction	No	Good clinical outcome
Frunza-Stefan et al. [11]	5000	257	Positive/Positive	Yes, normal, VDRL negative, PCR not performed	Yes, immunostaining positive, PCR not performed	No	No	three weekly doses of injection benzathine penicillin 2.4 MU	Yes, non-steroid anti-inflammatory drugs	Good clinical outcome
Braue et al. [4]	NR*1	450	Positive/Positive	Yes, normal, serology negative, PCR not performed	Yes, immunostaining negative, PCR not performed	Biopsy of the terminal ileum and of a rectal mass and lymph node biopsies	Yes, CT	IV penicillin G 24 MU daily for two weeks and an additional three weekly doses of injection benzathine penicillin 2.4 MU	No	Good clinical outcome
Khatri et al. [12]	130	154	NR/Positive	Yes, pleocytosis VDRL negative PCR not performed	Yes, Immunostaining negative, PCR not performed	No	No	Treated for neurosyphilis, (CSF pleocytosis), therapy not indicated	No	Good clinical outcome
Vasudevan et al. [9]	10,000	196	Positive/Positive	Yes normal, VDRL negative, PCR not performed	Yes, immunostaining and PCR not performed	No	No	Single dose of 2.4 MU of benzathine penicillin	No	Good clinical outcome
Bernal et al. [13]	< 50	280	Positive/Positive	Yes, normal, VDRL negative, PCR not performed	No	Puncture of the aqueous humor	Yes, CT	2 g of IV ceftriaxone for 2 weeks	No	Slight visual impairment
Hardwick et al. [14]	NR	NR	Positive/Positive	No	No	No	No	Injection of procaine penicillin for 17 days	Yes, Montelukast 10 mg daily for 5 months	Good clinical outcome
Moloney et al. [15]	NR	340	NR/Positive	Yes, pleocytosis and elevated protein, VDRL < 1:2, PCR negative	No	Vitreous biopsy, PCR positive	Yes, CT and MRI	IV benzylpenicillin 2.4 g 4-hourly for 14 days	No	Good clinical outcome
Brochard et al. [16]	1000	329	Positive/Positive	Yes, normal, VDRL negative, PCR not performed	Yes, immunostaining positive and PCR negative	Joint Aspiration, PCR positive	No	IV benzylpenicillin for 14 days	No	Good clinical outcome
Ceccarelli et al. [17]	0	227	Positive/Positive	Yes, normal, serology negative, PCR not performed	No	No	Yes, MRI	IV penicillin G 2.4 MU for 14 days and an additional three weekly doses of injection benzathine	Yes, Prednisone 25 mg/die for 14 days	Left eye vision was lost and right visual acuity was 7/10
								penicillin 2.4 MU		
Rushing et al. [5]	< 50	43	Positive/Positive	Yes, Normal, VDRL negative, PCR negative	No	Brain biopsy	Yes, MRI	IV penicillin G for 14 days	No	Good clinical outcome
Bucher et al. [6]	922	177	Positive/Positive	Yes, pleocytosis and increased CSF total protein levels, VDRL reactive, PCR not performed	No	No	Yes, CT and MRI	IV penicillin G 24 MU daily for two weeks and an additional three weekly doses of injection benzathine penicillin 2.4 MU	No	After one-year neurologic examination showed mild left-sided weakness
Our case	42	318	Positive/Positive	Yes, pleocytosis and increased CSF total protein levels, RPR negative, PCR negative	No	No	Yes, CT and MRI	IV penicillin G 2.4 MU for 14 days	No	Slight visual impairment

^*^^1^not reported

We classified 10 cases as “unmasking syphilis” and four as “paradoxical syphilis”. The median time for the development of IRIS after the beginning of ART was 28 days (IQR 13 to 46.5 days).

Skin and ocular manifestations were the most frequently described; all cases were compatible with secondary syphilis, and there were genital lesions in three.

A diagnosis of neurosyphilis/ocular syphilis was posed in 8 cases: six were characterized by ocular involvement and two by a neurological deficit. Ocular manifestations are diversified, and any component of the eye can be involved. The mostly disorders described were the reduction of visual acuity, scotomas, blurred vision, floaters, conjunctival injection, tearing, and eye pain. Neurological involvement was only in two cases with motor, language, and memory deficits appearance [5, 6]. Skin manifestations were heterogeneous and sometimes atypical. Braue et al describe a case of malignant syphilis characterized by necrotic warty lesions with a tumoral appearance whose histology initially set cutaneous lymphoma in the differential diagnosis [4]. Skin manifestations were associated with ocular involvement in four cases. The frequent ocular manifestation in these patients can be justified by immunocompromising that promotes the rapid spread of the pathogen. Lumbar puncture was performed in most cases, and CSF alterations were in a few, predominantly pleocytosis. Negative syphilis serology and or polymerase chain reaction usually was observed like in our case. Nevertheless, the absence of CSF alterations should never exclude neurosyphilis when clinical manifestations are suggestive. In all cases, the patients were not on ART either because they were naïve or because of inadequate adherence to therapy. We didn’t observe a particular ART regimen associated with IRIS syphilis, but most therapies were INSTI or protease-inhibitor based. ART induces restoration of a cellular immune response against Treponema pallidum antigens and may probably result in the progression toward exuberant clinical features of the disease [2]. In almost all cases, basal serology for syphilis was negative and became positive after IRIS manifestation. It can happen in an HIV-positive patient with less than 200 CD4+T cells because a humoral response does not develop or could be suppressed due to the dysfunction of CD4+T-cells [7]. It is plausible that the restoration of immune responses as a result of effective HIV-1 treatment triggers the unmasking of subclinical Treponema pallidum infection and subsequent seroconversion. On the other hand, a negative syphilis serology could be caused by the prozone phenomenon or the hook effect, in which an overabundance of an antigen led to a false-negative result [3, 8–10]. Treatment for IRIS syphilis does not differ from standard syphilis therapy, and steroid therapy would seem unnecessary. Systemic steroid use has been reported for a short period in a few cases.

In conclusion, our case and the others reported in the literature suggest that IRIS associated with syphilis should be considered when an unusual rash or ophthalmologic compromise appears after the ART initiation. A negative syphilis serology before beginning antiretroviral therapy could convey the impression that syphilis has been ruled out; a high index of suspicion should be maintained instead when symptoms suggestive of syphilis are noticed after treatment has begun.

## Data Availability

The datasets used and/or analysed during the current study are available from the corresponding author on reasonable request.

## Abbreviations

IRIS: Immune reconstitution inflammatory syndrome
IV: Intravenous
HIV: Human immunodeficiency virus
RPR: Rapid plasma reagin
CT: Computer tomography
MRI: Magnetic resonance imaging
CSF: Cerebrospinal fluid
RNA: Ribonucleic acid
AIDS: Acquired immunodeficiency syndrome
CDC: Centers for Disease Control
IQR: Interquartile range
INSTI: Integrase Strand Transfer Inhibitor
